# SARS-COV-2 Variants: Differences and Potential of Immune Evasion

**DOI:** 10.3389/fcimb.2021.781429

**Published:** 2022-01-18

**Authors:** Sandro M. Hirabara, Tamires D. A. Serdan, Renata Gorjao, Laureane N. Masi, Tania C. Pithon-Curi, Dimas T. Covas, Rui Curi, Edison L. Durigon

**Affiliations:** ^1^ Interdisciplinary Program of Health Sciences, Cruzeiro do Sul University, São Paulo, Brazil; ^2^ Department of Molecular Pathobiology, New York University, New York, NY, United States; ^3^ Butantan Institute, São Paulo, Brazil; ^4^ Ribeirão Preto Medical School, University of São Paulo, Ribeirão Preto, Brazil; ^5^ Immunobiological Production Section, Bioindustrial Center, Butantan Institute, São Paulo, Brazil; ^6^ Laboratory of Clinical and Molecular Virology, Department of Microbiology, Institute of Biomedical Sciences, University of São Paulo, São Paulo, Brazil; ^7^ Scientific Platform Pasteur University of São Paulo, São Paulo, Brazil

**Keywords:** COVID-19, variant of concern, neutralizing antibody, vaccines, immune escape, delta variant, omicron variant

## Abstract

The structural spike (S) glycoprotein of severe acute respiratory syndrome coronavirus-2 (SARS-CoV-2) plays an essential role in infection and is an important target for neutralizing antibody recognition. Mutations in the *S* gene can generate variants of concern (VOCs), which improve “viral fitness” through selective or survival advantages, such as increased ACE-2 receptor affinity, infectivity, viral replication, higher transmissibility, resistance to neutralizing antibodies and immune escape, increasing disease severity and reinfection risk. Five VOCs have been recognized and include B.1.1.7 (U.K.), B.1.351 (South Africa), P.1 (Brazil), B.1.617.2 (India), and B.1.1.529 (multiple countries). In this review, we addressed the following critical points concerning VOCs: a) characteristics of the SARS-CoV-2 VOCs with mutations in the *S* gene; b) possible evasion of variants from neutralizing antibodies generated through vaccination, previous infection, or immune therapies; c) potential risk of new pandemic waves induced by the variants worldwide; and d) perspectives for further studies and actions aimed at preventing or reducing the impact of new variants during the current COVID-19 pandemic.

## Introduction

Severe acute respiratory syndrome coronavirus-2 (SARS-CoV-2) is a single-stranded positive-sense RNA virus containing a genome with 29,903 nucleotides and 29 proteins (Focosi and Maggi, 2021). The virus has six major open-reading frames (ORFs): ORF1a, ORF1b, S (spike), E (envelope), M (membrane), and N (nucleocapsid), and several accessory ORFs: ORF3a/b, ORF6, ORF7a, ORF7b, ORF8, ORF9b/c, and ORF10 (Kim et al., 2020; Zhu et al., 2020; Finkel et al., 2021).

**Figure 1:**
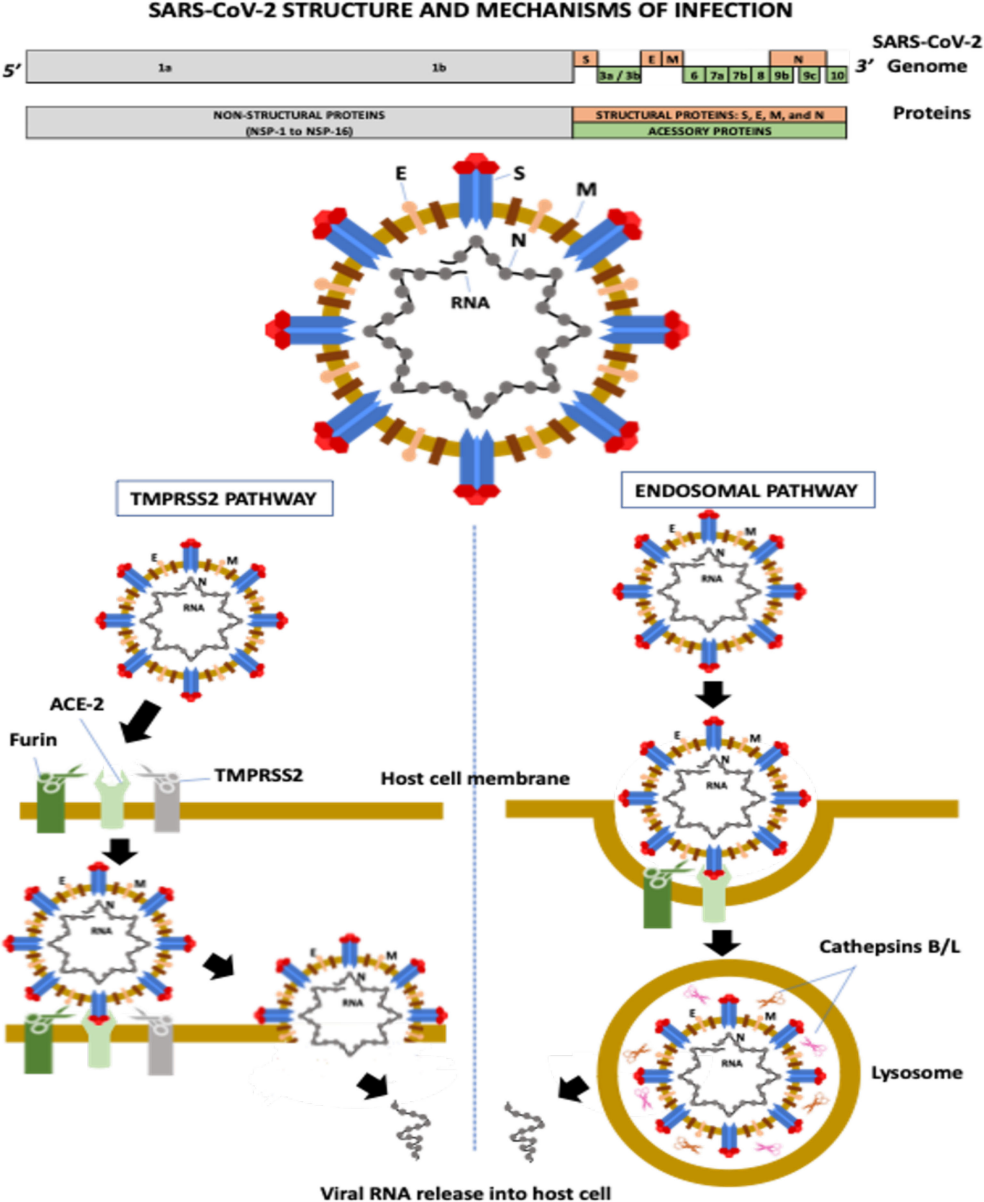
SARS-CoV-2 structure and mechanisms of infection. ACE-2, angiotensin-converting enzyme-2; E, envelope; M, membrane; N, nucleocapsid; NSP, non-structural protein; S, spike; TMPRSS-2, transmembrane serine protease-2.

ORF1a and ORF1b account for two-thirds of the SARS-CoV-2 genome. ORF1a encodes the polyprotein PP1a and the polyprotein PP1ab is a result of the overlapping translation of ORF1a and ORF1b. Both polyproteins (PP1a and PP1ab) are cleaved into 16 nonstructural proteins (NSPs 1 to 16): NSP1 (leader protein), NSP2 (unknown function), NSP3 (papain-like proteinase), NSP4 (transmembrane nsp containing four transmembrane domains and one luminal domain), NSP5 (3C-like proteinase), NSP6 (putative transmembrane nsp containing six transmembrane domains and two small luminal domains), NSP7 and NSP8 (the NSP7-NSP8 heterodimer interacts with the NSP12 forming the RNA polymerase complex), NSP9 (RNA-binding protein), NSP10 (cofactor for nsp14 and nsp16), NSP11 (unknown function), NSP12 (RNA-dependent RNA polymerase, RdRp), NSP13 (helicase), NSP14 (3′ to 5′ Endonuclease, N7‐Methyltransferase), NSP15 (endoribonuclease, NendoU), and NSP16 (2′‐O‐Ribose‐Methyltransferase) (Snijder et al., 2016; Finkel et al., 2021). ORFs S, E, M, and N encode four structural proteins, whereas accessory ORFs lead to the formation of several accessory proteins (Kim et al., 2020) (**Figure 1**).

The M protein is the most abundant transmembrane protein and is associated with virus assembly and morphology. The E protein also participates in virus assembly, release, and ion channel activity processing. In coronaviruses, ion channel activity has been implicated in viral infectivity. The N protein encapsulates the viral RNA and, along with NSPs, plays a crucial role in virus replication, transcriptional processes, and genome assembly (Nieto-Torres et al., 2014; Abdel-Moneim et al., 2021).

The S glycoprotein is a homotrimer, and each monomer contains two subunits, S1 and S2. S1 contains the N-terminal domain (NTD) and the receptor-binding domain (RBD), which recognize and bind to the angiotensin-converting enzyme-2 (ACE-2) receptor required for virus attachment and entry into host cells (Ou et al., 2020; Abdel-Moneim et al., 2021). The RBD, precisely the receptor-binding motif (RBM) region, also contains the main antigenic epitopes recognized by neutralizing antibodies (nAbs) (Abdel-Moneim et al., 2021). S2 has several domains and mediates membrane fusion between the viral envelope and the host cell (Abdel-Moneim et al., 2021). The S protein is highly N-glycosylated at at least 22 sites: 13 in S1 and nine in S2 (Yao et al., 2020). Two main RBD conformations have been described, standing-up and lying-down states, with high and low affinity to ACE2, respectively (Yao et al., 2020). Although RBD of SARS-CoV-2 presents a higher affinity to ACE2 than the RBD of SARS-CoV, most RBD in the entire SARS-CoV-2 is in the lying-down state, resulting in a similar or even lower affinity to the receptor than SARS-CoV (Yao et al., 2020). The exposure of N-linked glycans is modified according to the RBD conformation (10 in the RBD-down and 7 in the RBD-up states), suggesting that these molecules can participate in the interaction between SARS-CoV-2 and the host cell (Yao et al., 2020).

The first step of viral infection is RBD binding to ACE2 on the host cell. Several proteases then help S glycoprotein cleavage, including transmembrane serine protease-2 and -4 (TMPRSS-2 and -4), furin-like enzymes, and endosomal cathepsins B/L (Shang et al., 2020) (**Figure 1**). Cleavage is required for S protein priming and activation, allowing the membrane fusion process and viral RNA entry into a host cell (Hoffmann et al., 2020; Zang et al., 2020). The activity of these proteases is associated with increased transmissibility, virulence, and cell and tissue tropism (Abdel-Moneim et al., 2021).

Furin is a serine protease involved in the preactivation of the S protein, which enhances virus entry mainly in host cells with low expression of other proteases, including TMPRSS2 and cathepsins (Shang et al., 2020). The protease recognizes the furin-like cleavage site, a multibasic site composed of three arginine molecules and one alanine (RRAR) located at the S1-S2 junction. After this cleavage preactivation of the S protein (S1-S2 junction), a second cleavage site located in the S2 subunit (S2′ site) is critical for membrane fusion during virus entry (Takeda, 2022). The cleavage of the S2’ site occurs by two pathways: a) the TMPRSS2 pathway and b) the endosomal pathway (Hoffmann et al., 2020) (**Figure 1**). TMPRSS2 is a serine protease with trypsin-like endopeptidase activity located at the cell surface that promotes priming and activation of the S protein, allowing the interaction of the S2 fusion peptide (FP) domain with the host cell membrane, consequently leading to membrane fusion and viral RNA release into the host cytosol (Kielian, 2020).

The second SARS-CoV-2 infection mechanism occurs through the endosomal pathway, in a process that depends on phosphatidylinositol-3-phosphate-5-kinase activity, required for the synthesis of phosphatidylinositol-3,5-biphosphate (PI-3,5-P2), which is critical for endosome maturation, and on two-pore channel subtype 2 (TPC2), present in late endosomes and lysosomes, which is the main downstream effector of PI-3,5-P2, mediating cation transport, mainly Na^+^ and Ca^2+^ (Ou et al., 2020). Inhibition of the TPC2 activity or PI-3,5-P2 production prevents SARS-CoV-2 endocytosis (Ou et al., 2020). In lysosomes, cysteine proteases (cathepsins B and L) promote S protein cleavage at the S2’ site, allowing the interaction and fusion of the viral envelope and the lysosomal membrane and, consequently, the viral nucleocapsid is released into the host cytosol (Shang et al., 2020).

Any disturbance in the S protein structure can modulate one or more processes involved in the viral infection and eventually provide some selective advantage for the virus (Harvey et al., 2021). For instance, a specific mutation (or a combination of mutations) in the *S1* or *S2* subunits can modify: a) the affinity of S1 RBD to ACE2, increasing the virus binding to the host cells; b) the number and exposition of glycosylated sites, facilitating the interaction/accessibility between the viral envelope and host cell plasma membrane; c) the percentage of lying-down and standing-up states of the S1 RBD, elevating the general virus affinity to ACE2; d) the affinity of cleavage sites for proteases, improving the membrane fusion process; and e) recognition by nAbs, reducing the humoral immune response and inducing immune evasion.

The humoral response against SARS-CoV-2 involves specific nAbs against viral epitopes, mainly against the S glycoprotein (Ni et al., 2020). Epitopes of the N protein are highly conserved among different coronaviruses, inducing cross-reacting antibody generation. However, nAbs against the S protein protect against SARS-CoV-2 infections (Melenotte et al., 2020). Therefore, nAb activity against S glycoprotein allows for the evaluation of the responses induced by vaccines, convalescent plasma, and antibody therapies, as well as the potential immune evasion by different VOCs. The emergence of new variants at the end of 2020 raised new concerns about viral fitness and antibody-based therapies, including vaccines, convalescent sera, and monoclonal antibodies (mAbs) (Fontanet et al., 2021). In addition, new waves of the COVID-19 pandemic have been attributed to the new variants in several parts of the world (Fontanet et al., 2021).

In this review, we address the following critical points concerning SARS-CoV-2 variants: the characteristics of the variants with concerning mutations in the spike gene; the possible evasion of VOCs from nAbs generated through vaccination or previous infection; and perspectives for further studies and actions aimed at preventing or reducing the impact of new VOCs on the COVID-19 pandemic.

## SARS-CoV-2 Spike Gene Mutations

Several genes of SARS-CoV-2, including *S*, *N*, and *NSP12* (RdRP), present a high mutational range (Focosi and Maggi, 2021). However, compared to other RNA viruses, coronaviruses present a low mutational frequency due to NSP14, which exhibits 3′ to 5′ Exonuclease (ExoN) activity that is critical for high viral replication fidelity (Smith et al., 2014). It has been suggested that other factors can increase the number of mutations in SARS-CoV-2.

For example, control measures have had a sizeable negative impact on the economy. Most countries adopted incomplete or insufficient preventive/restrictive measures, with partial participation/adherence, resulting in ineffective control of the COVID-19 pandemic. Consequently, virus transmission and spread increased the probability of new mutations, leading to the emergence of variants with selective advantages (Chen and Lu, 2021). Additionally, individuals with impaired immune competence suffer from prolonged SARS-CoV-2 infections, which increases the likelihood of new mutations (Choi et al., 2020). There have also been many SARS-CoV-2 reinfections reported, raising immune pressure and selecting mutations that potentially can help escape immune defense (Abdel-Moneim et al., 2021). Lastly, viral adaptation in susceptible animals and subsequent human infection can produce additional mutations in SARS-CoV-2 (Abdel-Moneim et al., 2021).

Mutations can occur in any region of the SARS-CoV-2 genome. Most mutations are silent, meaning that they do not modify the primary amino acid sequence, the function of the translated proteins or viral infectivity. However, a single mutation, or a combination of mutations, can yield variants with selective and survival advantages and improved viral fitness. These mutational variants can present increased infectivity and/or transmissibility, human ACE-2 receptor binding affinity, viral replication, pathogenicity and reinfection risk. Moreover, depending on the location of the mutation, changes in antigenicity and host-, vaccine- or mAb-induced immune response evasion with alteration in crucial epitopes recognized by nAbs and/or decreased T cell immunity (Dearlove et al., 2020; Abdel-Moneim et al., 2021; Altmann et al., 2021; Callaway and Ledford, 2021; Focosi and Maggi, 2021).

While mutations in other genes could generate variants with enhanced viral infectivity, replication, and immune escape potential (Khateeb et al., 2021; Nguyen et al., 2021), this review will focus on *S* gene mutations because the spike protein is the most extensively studied viral infection protein and the main protein target for vaccine development. For instance, recent studies have reported that R203K/G204R modifications in the N protein are associated with high viral replication, infectivity, and transmissibility in cellular and animal models (Wu et al., 2021; Zhu et al., 2021b); it has been observed that these modifications appear at a high prevalence in the B.1.1.7 variant (Collier et al., 2021). NSP1 protein is important for suppressing interferon I signaling and increasing viral replication (Xia et al., 2020; Lin et al., 2021). Notably, Lin et al. (2021) observed that a deletion in NSP1 (Δ500-532) is related to low plasma IFN-β levels and viral load. The high prevalence of the P323L mutation in NSP12 (RdRp) has been implicated in viral replication (Koyama et al., 2020).

Mutations in ORF8 have been suggested to augment viral transmission and immune evasion potential because its gene products participate in the RNA polymerase complex and are involved in controlling the host cells’ major histocompatibility complex class I (Young et al., 2020; Flower et al., 2021; Pereira, 2021). Pereira (2021) observed a high prevalence of a premature stop codon at position 27 in ORF8 (Q27stop) that occurs in the B.1.1.7 variant, potentially contributing to its high transmission rate and spread.

Emerging variants can be considered a variant under investigation (VUI), a variant of interest (VOI), or a variant of concern (VOC). The WHO has recognized several VOIs, including B.1.427 and B.1.429 from the USA (California, WHO alert since July 6, 2021), B.1.525 from the United Kingdom and Nigeria, B.1.526 from the USA (New York), B.1.617.1 and B.1.617.3 from India, P2 from Brazil, and C.37 from Peru. Furthermore, the WHO has classified five variants as VOCs: B.1.1.7 from the United Kingdom (501Y. V1, VOC 202012/01, alpha variant), B.1.351 from South Africa (501Y. V2, VOC 202012/02, beta variant), P.1 from Brazil (501Y. V3, VOC 202101/02, gamma variant); B.1.617.2 from India (VOC 202104/02, delta), and B.1.1.529 from multiple countries (omicron variant). Notably, the B.1.617.2 variant was linked to the fast spread of SARS-CoV-2 in several countries (Adam, 2021).

The D614G mutant in the *S* gene, first identified in Europe in January 2020, was one of the first SARS-CoV-2 mutations to spread worldwide (Conti et al., 2021; Dearlove et al., 2020). This mutation is positioned between the S1 and S2 subunits (Dearlove et al., 2020) and has been reported to increase *in vitro* viral infectivity (Dearlove et al., 2020; Korber et al., 2020; Groves et al., 2021), affinity binding to the ACE-2 receptor and transmissibility (Volz et al., 2020; Ozono et al., 2021), protease-induced S protein cleavage (Gobeil et al., 2021), replication, and viral loads (Abdel-Moneim et al., 2021). Despite the apparently enhanced “viral fitness” (Dearlove et al., 2020; Plante et al., 2020) and its ability to neutralize the activity of antibodies induced by previous infections or vaccines (Dearlove et al., 2020; Groves et al., 2021), the clinical outcomes or pathogenicity remain unchanged (Volz et al., 2020; Groves et al., 2021). It has been proposed that the D614G mutation causes the ACE-2 receptor to assume “open conformation”, increasing the binding affinity (Yurkovetskiy et al., 2020) and the virus’s susceptibility to nAbs (Garcia-Beltran et al., 2021a).

The B.1.1.298 variant (mink Cluster 5) was one of the first to contain the D614G mutation. This variant was associated with an outbreak on Denmark mink farms (Oude Munnink et al., 2021), resulting in 17 million Danish minks being culled as a preventive measure to stop virus evolution and spread (Garcia-Beltran et al., 2021b). It has been suggested that other modifications, including Y453F in the RBD of the S protein, P323L in NSP12 (a component of RdRp), and R203K and G204R in the N protein, also contributed to the improved viral fitness of the B.1.298 variant (Plante et al., 2021). Notably, the D614G mutation has become more predominant, appearing in all recently identified variants.

The first VOC described (VOC 202012/01) was the B.1.1.7 lineage (20I/501Y. V1), identified in the United Kingdom (Sep 2020). This variant is now present on all continents. In December 2020, B.1.1.7 was responsible for one-quarter of the total COVID-19 cases worldwide and two-thirds of the cases in the United Kingdom (Conti et al., 2021). Compared to the original virus, B.1.1.7 exhibits a 40–70% increase in transmissibility (Graham et al., 2021; Volz et al., 2021). The B.1.1.7 lineage has 23 mutations in the *S*, *N*, and *ORF-8* genes, but the impact of each mutation on viral fitness and survival or vaccine efficacy is not completely known (Conti et al., 2021; Focosi and Maggi, 2021).

The S protein of the B.1.1.7 lineage contains several amino acid mutations, including D614G and N501Y, and deletions ΔH69/ΔV70 (Focosi and Maggi, 2021). The S RBD N501Y mutation increases the binding affinity to the ACE-2 receptor and transmissibility (Starr et al., 2020; Graham et al., 2021; Focosi and Maggi, 2021). Gu et al. (2020) developed a mouse-adapted strain model (MASCp6) to evaluate the SARS-CoV-2 infectivity and virulence after intranasal inoculation and observed that the N501Y mutation favors interaction with ACE2 and promotes virus entry, consequently leading to enhanced virulence. Recent studies have suggested that the N501Y mutation has a low impact on clinical outcomes and pathogenicity (Conti et al., 2021; Graham et al., 2021) and the immune response generated by mAbs, vaccines, or previous infection (Muik et al., 2021; Focosi and Maggi, 2021). However, Davies et al. (2021) evaluated more than 2.2 million people with SARS-CoV-2 positive tests and 17,452 related deaths in England and observed a 61% higher risk of death risk in those infected with the B.1.1.7 variant than other pre-existing variants. Thus, the B.1.1.7 variant presents increased transmissibility and disease severity.

The B.1.351 lineage (20H/501Y. V2, VOC 202012/02) emerged in South Africa (Oct 2020), probably favored by the high immune pressure, and spread to other African countries, Asia, Australia, and North and Central America (Focosi and Maggi, 2021). By the end of 2020, this variant was responsible for more than 90% of all COVID-19 cases in South Africa (Callaway and Mallapaty, 2021). This lineage has several structural and nonstructural mutations, including three critical mutations in the RBD of the S protein (K417N, E484K, and N501Y) that seem to play a crucial role in the improved “viral fitness” and survival adaptations compared to the other strains in some regions where it was prevalent (Focosi and Maggi, 2021). The K417N mutation exacerbated immune escape from nAbs and reduced vaccine effectivity against infection (Callaway and Mallapaty, 2021; Focosi and Maggi, 2021), and the E484K modification is associated with increased binding to the ACE-2 receptor (Focosi and Maggi, 2021) and a decreased or even abrogated response to Ab neutralization induced by previous infection, vaccination, or monoclonal Ab therapy (Liu Z. et al., 2021). Furthermore, five mutations in the NTD of the *S* gene were proposed to contribute to improved viral microenvironment adaptations (Li et al., 2021). This lineage is also associated with increased spreading (Garcia-Beltran et al., 2021a) and reinfection cases in subjects previously infected with the original SARS-CoV-2 (Staub et al., 2021).

**Figure 2:**
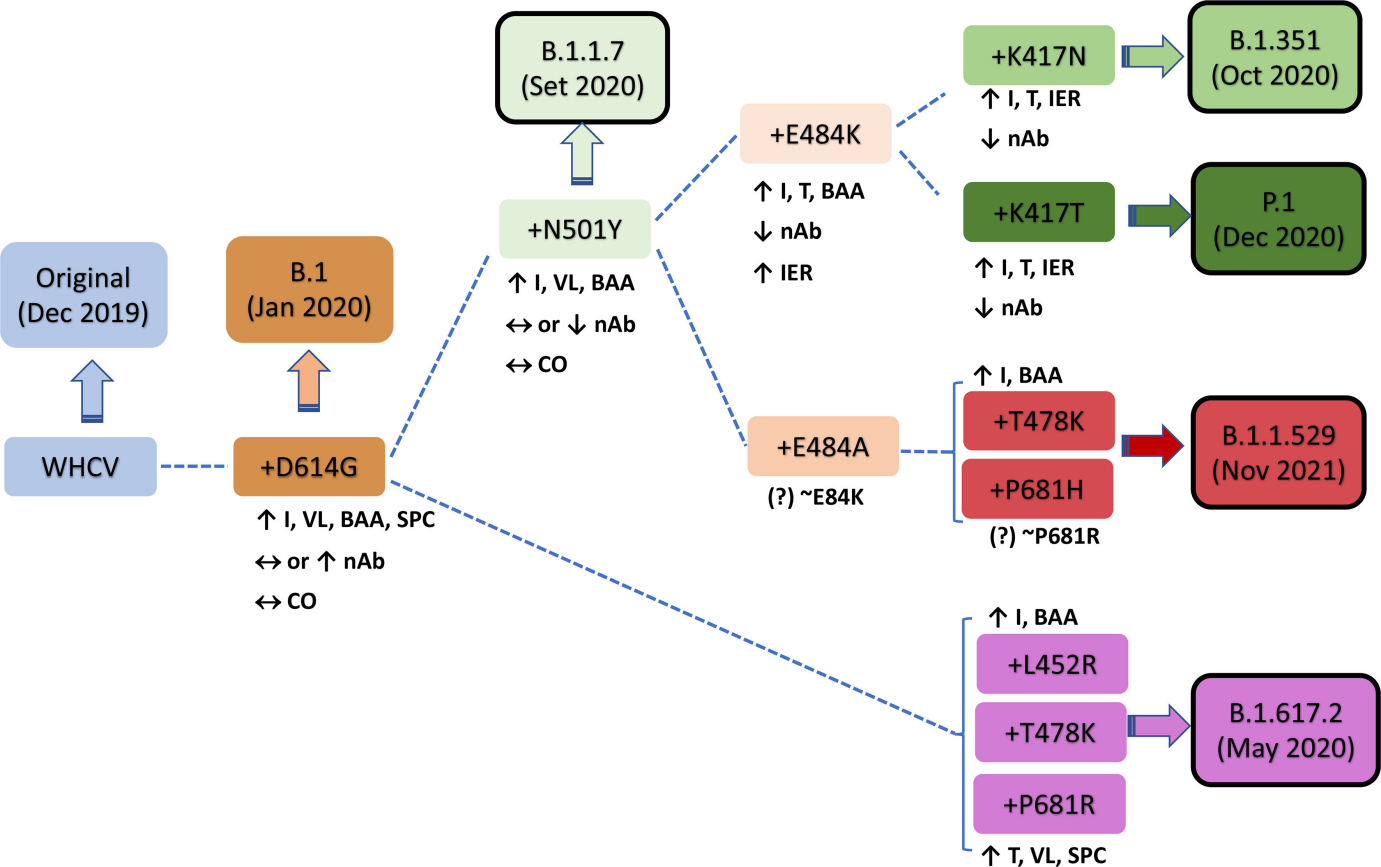
Characteristics of key mutations in variants of concern (VOCs). A comparison was performed versus the original virus (WHCV) or the B.1 strain (+D614G). BAA, binding affinity to ACE2; CO, clinical outcome; I, infectivity; IER, immune evasion risk; nAb, neutralizing antibody; SPC, spike protein cleavage; T, transmissibility; VL, viral load; WHCV, WH-Human 1 coronavirus.

**Figure 3:**
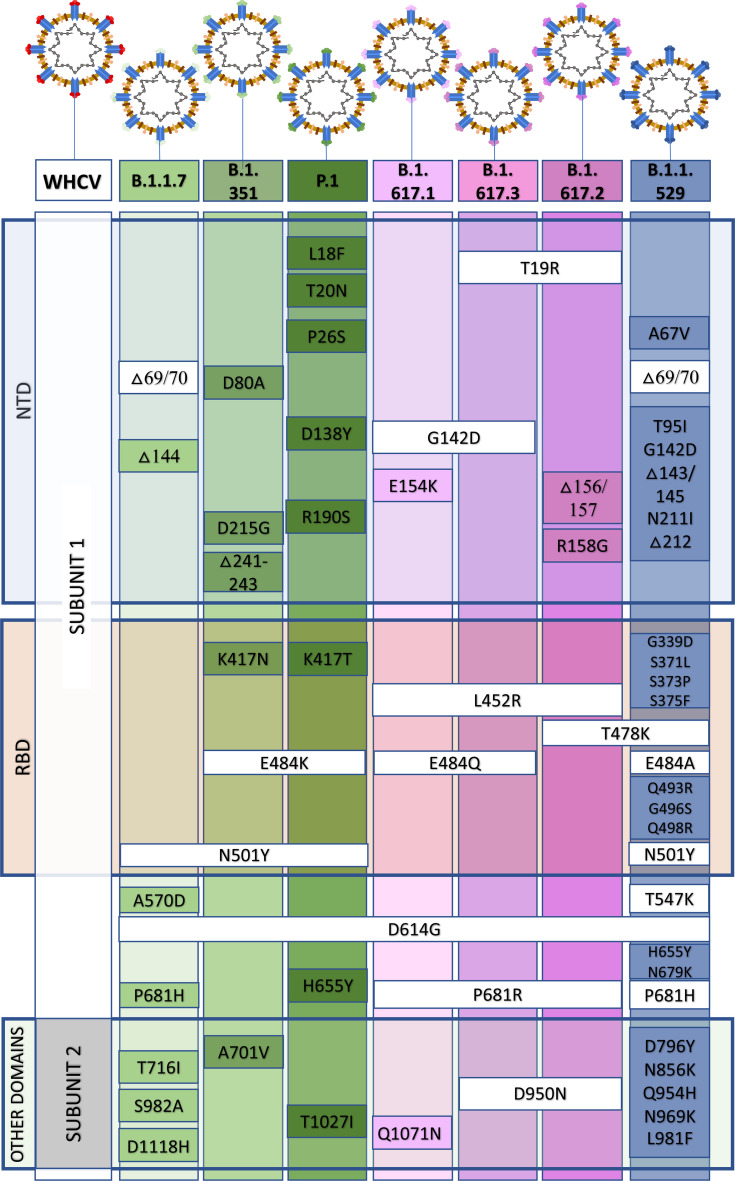
Similar and differential mutations in the spike (S) protein from the B.1.1.7, B.1.351, P.1, B.1.617, and B.1.1.529 variants of concern. NTD, N terminal domain; RBD, receptor-binding domain.

In December 2020, the P.1 lineage (20J/501Y. V3, VOC 202101/02, also called P.1) accounted for 42% of the total cases in Manaus, Brazil (Chen and Lu, 2021), and in February 2021, it was discovered in Japan in samples from individuals traveling from Manaus. The three main mutations are the same as in B.1.351: K417T, E484K, and N501Y (Focosi and Maggi, 2021). This lineage has increased ACE-2 receptor binding affinity, transmissibility (Focosi and Maggi, 2021; Francisco et al., 2021), infectivity in mice (Chen and Lu, 2021), resistance to immune response (Focosi and Maggi, 2021; Francisco et al., 2021), and risk of reinfection (Chen and Lu, 2021; Taylor, 2021; Sabino et al., 2021). The main similarities and differences among B.1.1.7, B.1.351, and P.1 are summarized in **Figure 2**. Similar and differential mutations of the main SARS-CoV-2 variants are shown in **Figure 3**.

The B.1.617 lineage contains three sublineages: B.1.617.1, B.617.2 (delta variant), and B.1.617.3. B1.617.2 exhibits higher transmissibility than the ancestral strain, and studies suggest a high risk of hospitalization compared to the original strain or the B.1.1.7 variant (Liu and Rocklöv, 2021; Ong et al., 2021; Sheikh et al., 2021). In a short review, Liu and Rocklöv (2021) reported a basic reproductive number (*R*_0_) of 5.08 for the delta variant versus 2.79 for the ancestral SARS-CoV-2 strain. Since no difference was observed in the median age and disease duration between patients infected with B.1.617.2 or non-B.1.617.2 strains (Mlcochova et al., 2021), the elevated risk of hospitalization is probably due to the high transmissibility of the B.1.627.2 variant compared to other strains. Most fully vaccinated people are protected against the B.1.617 variants (Lopez Bernal et al., 2021). However, even after full vaccination, people can be infected by B.1.617.2 or other variants and transmit them to others, albeit with a lower risk of disease severity and shorter infection period than unvaccinated individuals (Ong et al., 2021; Sheikh et al., 2021). Notable mutations in the B.1.617.2 variant include L452R, T478K and E484Q in the S RBD and P681R in the cleavage site between S1 and S2.

The L452R mutation appears to increase the interaction between RBD and the ACE-2 and infectivity (Kirola, 2021). Moreover, the T478K mutation, together with L452R, helps stabilize the RBD–ACE-2 complex and elevate the virus infectivity rate (Cherian et al., 2021). The E484Q mutation enhances binding affinity to ACE-2 and potentially reduces antibody binding affinity, an observation similar to the E484K mutation (Kirola, 2021). In addition, the P681R mutation, located at the cleavage site between S1 and S2, has been associated with augmented transmissibility and viral load (Lopez Bernal et al., 2021). In a preprint work, Liu et al. (bioRxiv [Preprint]. 2021 Sep 5:2021.08.12.456173) observed that P681R modification leads to the increased furin cleavage site (S1-S2 junction), resulting in higher infectivity than the B.1.1.7 strain. The combination of mutations in the B.1.617.2 variant (delta variant) seems to impart the virus a selective advantage compared to the original virus and other variants, as evidenced by high transmissibility and infectivity, and potential immune evasion (Cherian et al., 2021; Kirola, 2021; Lopez Bernal et al., 2021).

Recently, an emerging SARS-CoV-2 variant was initially identified in South Africa, but it has been simultaneously detected in several other countries. On November 26, 2021, the WHO classified this variant as a VOC (B.1.1.529, omicron variant) because of the alarming epidemiological situation in South Africa (Callaway, 2021). The B.1.1.529 variant contains several mutations present in other variants, such as N501Y (alpha), E484A ~ E484K (beta and gamma), and T478K; P681H ~ P681R (delta). As discussed above, several of these changes observed in alpha, beta, and delta have been related to enhanced infectivity, transmissibility, and potential immune escape. However, it remains unclear whether or not the similarities with previous VOCs are related to the omicron variant’s rapid spread. In total, the B.1.1.529 (omicron) variant has more than 50 mutations, with more than 30 in the *S* gene alone (Callaway, 2021; GISAID, 2021). In addition, Wang and Cheng (2021) have identified potential mutations that can affect ACE2 and/or antibody binding. Omicron variant accumulates numerous mutations, including Q498R and S477N, which have been previously associated with elevated binding to ACE2 receptor, potentially enhancing viral infectivity to the host cells. Recently, it was observed a close connection between Omicron and the Alpha variants, suggesting that the omicron variant was circulating for an long period before its discovery (Kandeel et al., 2021). Ongoing research is trying to elucidate the role and effect of each mutation in the omicron variant. Currently, it appears as though the omicron variant does not increase disease severity or fatality (hospitalization and number of deaths), and there is no evidence of immune escape from approved vaccines.

In South Africa, the emergence of the omicron variant rapidly and concomitantly increased the number of daily cases from 273 cases/day on November 16 to more than 1,200 cases/day on November 25. Additionally, as of December 9, 2021, the omicron variant was confirmed in 63 countries in Africa, Europe, Australia, Asia and North, Central and South America (GISAID, 2021; Torjesen, 2021).

## Efficacy and Antibody Neutralization Activity of Vaccines Against SARS-CoV-2 Variants

Most COVID-19 vaccines use the S protein as the primary target, aiming to produce nAbs against the RBM regions, block the viral binding sites to the ACE-2 receptor in the host cells, and prevent infection (Chen R. E. et al., 2021). Since the first generation of vaccines was developed based on the original SARS-CoV-2 without S protein amino acid mutations (Chen R. E. et al., 2021; Dearlove et al., 2020), medical professionals now face the challenge of determining if the efficacy of these vaccines against the new variants is preserved or impaired (Chen R. E. et al., 2021; Dearlove et al., 2020). Presently, most approved COVID-19 vaccines protect against the described VOCs; however, constant surveillance and new studies about vaccine efficacies against the current VOCs and future SARS-CoV-2 variants globally are critical. It is also important to point out that most studies concerning nAbs activity were performed using SARS-CoV-2 pseudoviruses, which may not reflect the virus’s behavior in the real world. Furthermore, a reduction in nAb activity does not necessarily result in poor vaccine efficacy or effectiveness, as demonstrated by several recent studies.

For example, the mRNA-based BNT 162b2 vaccine (Pfizer/BioNTech) reached 95% efficacy against the original SARS-CoV-2 infection (Polack et al., 2020). Notably, using immune sera from vaccinated subjects, no difference (Kuzmina et al., 2021) or mild to moderately decreased nAb activity (1.7 to 6.0-fold) against the B.1.1.7 pseudovirus has been described (Collier et al., 2021; Hoffmann et al., 2021; Lustig et al., 2021; Muik et al., 2021; Supasa et al., 2021). Thus, this variant probably does not increase immune escape or attenuate vaccine efficacy (Muik et al., 2021; Supasa et al., 2021). In contrast, the nAb activity provided by the BNT 162b2 vaccine was significantly reduced (6.5 to 10.4-fold) or abrogated against the B.1.351 pseudovirus (Chen R. E. et al., 2021; Dejnirattisai et al., 2021; Hoffmann et al., 2021; Kuzmina et al., 2021; Lustig et al., 2021; Zhou et al., 2021). Additionally, a reduction of 2.1 to 5.1-fold and 1.4 to 3.0-fold in nAb activity was reported for the P.1 and B.1.617.2 variants, using the serum from vaccinated individuals (Dejnirattisai et al., 2021; Hoffmann et al., 2021; Liu J. et al., 2021; Lustig et al., 2021; Planas et al., 2021).

BNT 162b2 vaccine effectiveness was also evaluated in the Qatar population, when the B.1.1.7 and B.1.351 variants accounted for 50% and 44.5% of the total COVID-19 cases from February to March 2021 (Abu-Raddad et al., 2021). A mass vaccination campaign was performed in the country, with 385,853 people receiving one dose and 265,410 receiving two doses of the vaccine by the end of March 2021 (Abu-Raddad et al., 2021). After 14 days or more after the second dose of the BNT 162b2 vaccine, the effectiveness against B.1.1.7 variant infection was 89.5%, and 75.0% against B.1.351. Moreover, the vaccine’s effectiveness against severe COVID-19 cases or death was 97.4% against both variants (Abu-Raddad et al., 2021). In another real-world study performed in Qatar between December 2020 and September 2021, 950,232 people received at least one dose and 916,290 people two doses of the BNT162b2 vaccine (with an average of 21 days between doses) (Tang et al., 2021). The authors observed similar results against infection (74.3%) and severe/critical/fatal disease (92.7%) caused by B.1.351 variant and low effectiveness against B.1.617.2 infection (51.9%). Despite the reduced protection against infection, the vaccine was still highly effective against severe/critical/fatal disease (93.4%) caused by the B.1.617.2 variant.

Another mRNA-based vaccine, the mRNA-1273 vaccine (Moderna), also reached a high global efficacy of 94% and induced nAb production (Baden et al., 2021). Against the B.1.1.7 pseudovirus, no difference or a modest reduction in nAb activity was reported (Shen et al., 2021; Wu et al., 2021). On the other hand, a pronounced reduction in nAb activity (6.4-fold) against the B.1.351 variant was observed (Wu et al., 2021). Another study demonstrated that IgG antibody binding and neutralization activity are moderately impaired against the B.1.351 variant, but this vaccine is still efficient/effective against this variant (Edara et al., 2021b). Compared to B.1.351, the reduction in nAB activity was less pronounced in the P.1 and B.1.427/429 variants (Wu et al., 2021). The mRNA-1273 vaccine also exhibited reduced nAb activity (2.1 to 3.3-fold) against B.1.617.2 compared to the D614G strain (Choi et al., 2021). In the real-world study performed in Qatar described above (Tang et al., 2021), 564,468 people received at least one dose, and 509,322 received two doses of the mRNA-1273 vaccine (with an average of 28 days between doses). The authors reported 80.8% effectiveness against infection and 100% against severe, critical, or fatal disease caused by B.1.351 and 73.1% effectiveness against infection and 96.1% against severe, critical, or fatal disease caused by B.1.167.2 variant.

The recombinant spike protein-based NVX-CoV2373 vaccine (Novavax) presents an efficacy of 95.6% against the original SARS-CoV-2 strain (Callaway and Mallapaty, 2021; Moore and Offit, 2021). Against the B.1.1.7 pseudovirus, this vaccine has a low reduction in nAb activity (Shen et al., 2021), which is followed by a modest decrease in efficacy (85.6%) (Shen et al., 2021). In a South African phase 2a/b clinical trial, vaccine efficacy against mild to moderate disease was significantly reduced (49.4%) in a population of 4,387 participants when the B.1.351 variant was predominant (92.7%) (Shinde et al., 2021). In another study performed in South Africa, when more than 90% of the total COVID-19 cases were due to the B.1.351 variant (i.e., end of 2020 and the beginning of 2021), the vaccine reached an efficacy of 60% (https://www.novavax.com/sites/default/files/2021-02/20210202-NYAS-Novavax-Final.pdf). Currently, the B.1.617.2 variant is predominant in South Africa and worldwide, and data regarding effectiveness against this variant is crucial for understanding the real protection elicited by the Novavax vaccine.

The adenovirus vector-based ChAOx1-nCoV-19 vaccine (University of Oxford/AstraZeneca) was shown to have 66.7% efficacy against SARS-CoV-2 infection (Voysey et al., 2020). Against the B.1.1.7 pseudovirus vaccinee sera display reduced (9-fold and 2.5-fold) nAb activity without affecting vaccine efficacy (74.6%) in 499 infected people with the variant (Zhou et al., 2021; Supasa et al., 2021). Similar results were reported in another study comparing B.1.1.7 and non-B.1.1.7 lineages, with 70.4% and 81.5% efficacy against infection, respectively (Emary et al., 2021). For the B.1.351 lineage, the ChAOx-1nCoV-19 vaccine elicits less potent nAb production against the B.1.351 pseudovirus. B.1.351 is mainly characterized by the triple mutations in the RBD of the S protein and associated with reduced nAb titers (9-fold) and global efficacy against infection (10.4-20.4%) and impaired efficacy (21.9%) to prevent mild to moderate COVID-19 (Dejnirattisai et al., 2021; Madhi et al., 2021; Planas et al., 2021; Zhou et al., 2021). A small reduction in nAb activity was also reported for the P.1 (2.9-fold) and B.1.617.2 variants (5.0-fold) (Dejnirattisai et al., 2021; Planas et al., 2021). Notably, the real-world effectiveness against B.1.617.2 infection (67%) was similar to the wild-type (66.7%) and B.1.1.7 (74.6%) strains (Lopez Bernal et al., 2021).

The Ad26.COV2. S or JNJ-78436735 vaccine (Janssen), another adenovirus vector-based vaccine, has an efficacy of 72% against B.1.1.7 infection. The vaccine’s efficacy is reduced to 57% against the B.1.351 variant but is 89% effective at protecting against severe COVID-19 (https://www.jnj.com/johnson-johnson-announces-single-shot-janssen-covid-19-vaccine-candidate-met-primary-endpoints-in-interim-analysis-of-its-phase-3-ensemble-trial).

The vector-based Sputnik V vaccine or Gam-COVID-Vac (Gamaleya Institute) (Moore and Offit, 2021) presents 91.6% efficacy against SARS-CoV-2 infection (Logunov et al., 2021). However, the efficacies against B.1.1.7, B.1.351, and P.1 were reduced to 81%, 59%, and 52%, respectively. The serum nAb activity was not significantly altered against B.1.1.7 but was decreased against the B.1.1.351 (3.1-fold), P.1 (2.8-fold), and B.1.617.2 (2.5-fold) variants (Gushchin et al., 2021).

The Sinopharm and CoronaVac vaccines use inactivated virus-based technology. The CoronaVac vaccine was shown to be 83.5% (Tanriover et al., 2021) and 65.9% effective in studies conducted in Turkey and Chile, respectively (Jara et al., 2021). It was also demonstrated that nAb activity was unaffected against B.1.429 but was decreased against the B.1.1.7, B.1.351, and P.1 variants by 2.0, 5.2, and 3.9-fold, respectively (Chen Y. et al., 2021; Wang et al., 2021). For the Sinopharm vaccine, nAb activity was reduced against the B.1.1.7 (~2.0-fold) and B.1.351 (2.5 to 3.0-fold) variants (Wang et al., 2021).

More results related to the B.1.617.2 variant are necessary for all these vaccines to verify the real-world effectiveness against infection and severe or critical COVID-19 disease. Furthermore, a thorough analysis of this variant’s potential immune response evasion in vaccinated individuals must be conducted. In relation to omicron variant, there are some preliminary results from few studies with limited sample size suggesting that the incidence of virus reinfection in South Africa can be associated with humoral (antibody-mediated) immune evasion and nAb activity in vaccinated or previously infected individuals (Zhang et al., 2021).

Besides vaccines, convalescent sera have been used to evaluate the impact of variants on the nAb activity induced by the previous infection with the original SARS-CoV-2. Several studies observed a mild reduction (~2.9 to 3.0-fold) in nAb activity against the B.1.1.7 variant (Dejnirattisai et al., 2021; Supasa et al., 2021; Zhou et al., 2021). Partial (11 to 33 X). Variants containing the E484K mutation (e.g., B.1.351 and B.1.1.248 variants) were found to escape the immune response completely (Chen R. E. et al., 2021; Kuzmina et al., 2021; Zhou et al., 2021;14). Additionally, convalescent sera of individuals infected with the original SARS-CoV-2 displayed impaired or nonexistent IgG antibody binding and neutralization activity against the B.1.351 variant (4 to 8-fold; 13.3-fold) (Dejnirattisai et al., 2021; Edara et al., 2021a), persisting eight months post-infection (2.1-fold) (Edara et al., 2021b). Indeed, it has been estimated that 41.1 to 48% of convalescent sera are incapable of neutralizing the B.1.351 pseudovirus (Zhou et al., 2021; Wibmer et al., 2021). Furthermore, a similar reduction in nAb activity was observed against the P.1 and B.1.1.7 variants (3.1 and 2.9-fold, respectively) (Dejnirattisai et al., 2021).

A summary of the efficacy and nAb activity of the main vaccines against SARS-CoV-2 variants is presented in **Table 1**. In **Table 2**, we have provided the details of the protocols used in the studies.

**Table 1:** VOCs and vaccine-induced immune response resistance.

VOCs		WHCV (Wuhan/China)	B.1.1.7 UK	B.1.351 South Africa	P.1 Brazil	B.1.617.2 India
**Pfizer**	Efficacy	90.4-95.5%	89.5-93.7%*	75.0*%	N.D.	70-88%*
	NABs	–	↓ 0-3.3 X	↓ 3.3-16 X	↓ 2.2-6.7 X	↓ 2.1- 3.3 X
**Moderna**	Efficacy	94.1%	~	N.D.	N.D.	N.D.
	NABs	–	↓ 0-2.3 X	↓ 3-9 X	↓ 3.5-4.5 X	↓ 3 X
**AstraZeneca**	Efficacy	54-79%	70.4-74.5%*	10.4%	N.D.	67-77.3%*
	NABs	–	↓ 0-2.5 X	↓ 9 X	↓ 2.8-2.9 X	↓ 4.2-5 X
**Novavax**	Efficacy	89.3-95.6%	85.6%	49.4-60%	N.D.	N.D.
	NABs	–	↓ 2 X			
**Janssen**	Efficacy	66%	72%	57%	68.1%	N.D.
	NABs	–	↓ 2.8-3.3 X	↓ 5-10.6 X	↓ 3.3 X	
**Sputnik V**	Efficacy	91.6%	81%	59%	52%	N.D.
	NABs	–	↓ 0 X	↓ 3.1-3.5 X	↓ 2.8 X	↓ 2.5 X
**Sinovac**	Efficacy	65.9*-83.5%	N.D.	50%	N.D.	N.D.
	NABs	–	↓ 0 - 2.0 X	↓ 2.5 - 5.2 X	↓ 3.9 X	
**Sinopharm**	Efficacy	79.0-86%	N.D.	N.D.	N.D.	N.D.
	NABs	–	↓ 0-2.0 X	↓ 2.5 - 3.0 X		

NABs, neutralizing antibodies; N.D., not determined or under investigation; VOC, variant of concern; *: effectiveness evaluation instead efficacy analysis.

**Table 2:** Studies about efficacy/effectiveness and neutralizing antibody activity of vaccines and convalescent plasma against SARS-CoV-2 variants.

Study	Vaccine or plasma	Sample size	Methodology	Main findings
Abu-Raddad et al., 2021	BNT 162b2	>383,000 individuals with at least 1 dose and >265,000 with 2 doses; analysis >14 d after the 2nd dose	Effectiveness in a mass immunization campaign and virus sequencing of positive cases in Qatar	89.5% against B.1.1.7; 75.0% against B.1.351; 97.4% against severe disease
Alter et al., 2021	Ad6.COV2.S	25 adults at different vaccination regimens (14 d after the last dose)	Luciferase-based pseudovirus neutralizing antibody (psVNA) assay against WA1/2020, D614G, B.1.1.7, B.1.351, and P.1	↓ 2.8, 5-10.6, and 3.3 X in neutralizing B.1.1.7, B.1.351, and P.1 variants, respectively
Lopez Bernal et al., 2021	BNT 162b2	171,834 individuals: 96,371 unvaccinated; 51,470 vaccinated with 1 dose (analysis at >21 d); and 23,993 with 2 doses (analysis at >14 d)	Effectiveness by a test negative casecontrol design study and whole-genome sequencing in England	1 dose: 47.5% for B.1.1.7 and 35.6% for B.1.617.2
				2 doses: 93.7% for B.1.1.7 and 88% for B.1.617.2
	ChAdOx1-S			1 dose: 48.7% for B.1.1.7 and 30% for B.1.617.2
				2 doses: 74.5% for B.1.1.7 and 67% for B.1.617.2
Chen R. E. et al., 2021	BNT 162b2	10 vaccinee serum (one week after the 2nd dose)	FRNT using D614G wild-type, B.1.1.7, B.1.351, and B1.1.28 strains	↓ 2, 10, and 2.2 X in neutralizing B.1.1.7, B.1.351, and P.1
	Convalescent plasma	10 convalesdent plasma (30 d after infection)		↓ 2.5 X in neutralizing P.1
Chen Y. et al., 2021	CoronaVac	93 vaccinee serum (14 d after the 2nd dose)	Pseudovirus neutralization against different strains (Wuhan-1 wild-type, D614G, B.1.1.7, B.1.351, and P.1)	↓ 0, 5.2, and 3.9 X in neutralizing B.1.1.7, B.1.351, and P.1
Choi et al., 2021	mRNA-1273	8 vaccinee serum (7 d after the 2nd dose)	Pseudovirus neutralization against different strains (D614G, B.1.1.7, B.1.351, P.1, and B.1.617.2)	↓ 1.2, 6.9-8.4, 3.2, and 2.1-3.3 X in neutralizing B.1.1.7, B.1.351, P.1, and B.1.617.2
Collier et al., 2021	BNT 162b2	25 vaccinee serum (3 wks after the 2nd dose)	Pseudovirus neutralization against D614G strain and B.1.1.7 variant	↓ 1.9 X in neutralizing B.1.1.7
	Convalescent plasma	27 convalesdent plasma		↓ 4.5 X in neutralizing B.1.1.7
Dejnirattisai et al., 2021	BNT 162b2	25 vaccinee serum (4-14 d after the 2nd dose)	FRNT using Victoria and B.1.351 strains	↓ 3.3, 7.6, and 2.6 X in neutralizing B.1.1.7, B.1.351, and P.1
	AZD1222	25 vaccinee serum (14-2		↓ 2.5, 9, and 2.9 X in neutralizing B.1.1.7, B.1.351, and P.1
	Convalescent plasma	34 convalesdent plasma (4- 9 mo after infection)		↓ 2.9, 13.3, and 3.1 X in neutralizing B.1.1.7, B.1.351, and P.1
	BNT162b2	10 participants (7-27 d ater the 2nd dose)		↓ 3.3 X in neutralizing B.1.617.2
Edara et al., 2021a	mRNA-1273	15 participants (35-51 d ater the 2nd dose)	FRNT using WA1/2020 and B.1.617.2	↓ 3 X in neutralizing B.1.617.2
	Convalescent plasma	24 convalescent plasma (31-91 d after the onset of symptons)		↓ 2.4 X in neutralizing B.1.617.2
Edara et al., 2021b	mRNA-1273	19 participants (14 d after the 2nd dose)	IgG Ab binding by electrochemiluminescence- based multiplex immune assay	↓ 3.7 and 3.8 X Ab binding and virus neutralization (B.1.351)
	Acutely infected people	19 acutely infected participants (5-19 d after the onset of symptons)	Live virus neutralization using B.1 and B.1.351	↓ 4.4 and 3.3 X Ab binding and virus neutralization (B.1.351)
	Convalescent plasma	30 participants (1-3 and 3-8 mo after the onset of symptons)		↓ 4.4 and 3.3 X Ab binding and 4.8 and 2.1 X virus neutralization (B.1.351)
Emary et al., 2021	AZD1222	8,534 participants (1:1 AZD1222 vaccine vs meningococcal vaccine)	Clinical trial, phase 2/3, in the U.K.	70.4% efficacy against B.1.1.7 variant vs 81.5% efficacy against non-B.1.1.7 lineages
Geers et al., 2021	BNT 162b2	25 health care workers (2- 3 wks after the 1st dose and 3-4 wks after the 2nd dose)	PRNT assay against D614G strain and VOCs (B.1.1.7 and B.1.351)	↑ 2.5 and 2.2 X after the 1st and 2nd dose in neutralizing B.1.1.7
				↓ 2.7 and 3.3 X after the 1st and 2nd dose in neutralizing B.1.351
	Convalescent plasma	13 health care workers (3 wks after the onset of symptons)		↑ 2.8 X in neutralizing B.1.1.7 and ↓ 3X in neutralizing B.1.351
Gushchin et al., 2021	Sputnik V	27 vaccinee serum (30 d after the 2nd dose)	Virus neutralization against different strains (D614G, B.1.1.7, B.1.351, P.1, and B.1.617.2)	↓ 0, 3.1, 2.8, and 2.5 X in neutralizing B.1.1.7, B.1.351, P.1, and B.1.617.2
Hoffmann et al., 2021	BNT 162b2	15 donors, 13-15 d after the 2 nd dose	Entry of pseudotyped particles with different S protein strains (W.T., B.1.1.7, B.1.31, or P1) into target Vero cells	B.1.1.7: slight effect
				B.1.351 and P1: ↓ nAb activity
	Convalescent plasma	Individuals previously infected with WT SARSCoV-2		B.1.1.7: slight effect
				B.1.351 and P1: ↓ nAb activity
Kuzmina et al., 2021	BNT 162b2	10 vaccinee serum (21 d after the 1st dose or 9-11 d after the 2^nd^ dose)	Pseudovirus neutralization against wildtype strain or VOCs (B.1.1.7 and B.1.351)	No effect in neutralizing B.1.1.7
				↓ 6.8 X in neutralizing B.1.351
	Convalescent plasma	10 COVID19 recovered patients		↓ 1.5 X in neutralizing B.1.1.7
				↓ 6.8 X in neutralizing B.1.351
Liu C. et al., 2021	BNT 162b2	25 vaccinee serum (7-17 d after the 2^nd^ dose)	Live virus neutralization assay by FRNT using Victoria strain and VOCs (B.1.1.7, B.1.351, P.1, and B.1.617.2 variants)	↓ 3.2, 7.5, 2.6, and 2.5 X in neutralizing B.1.1.7, B.1.351, P.1, and B.1.617.2
	AZD1222	25 vaccinee serum (14-28 d after the 2^nd^ dose)		↓ 2.3, 9, 2.8, and 4.2 X in neutralizing B.1.1.7, B.1.351, P.1, and B.1.617.2
	Convalescent plasma	34 volunteers (4-9 wks after the infection)		↓ 2.9, 13.3, 3.1, and 2.6 X in neutralizing B.1.1.7, B.1.351, P.1, and B.1.617.2
Lustig et al., 2021	BNT 162b2	15-19 vaccinee serum (30 d after the 2^nd^ dose)	Neutralizing original (B.1) and VOCs strains (B.1.1.7, B.1.351, P.1, and B.1.617.2) and virus entry in VERO-E6 cells	↓ 1.7, 10.4, 2.3, and 2.1-2.6 X in neutralizing B.1.1.7, B.1.351, P.1 and B.1.617.2
Madhi et al., 2021	AZD1222	2,026 participants (1:1 AZD1222 vaccine or placebo)	Clinical, multicenter, double-blind, randomized trial, in the South Africa	21.9% efficacy against mild to moderate COVID-19
				10.4% efficacy against B.1.351
	ChAdOx1-S	10 vaccinee serum (after the 2^nd^ dose)	Pseudovirus neutralization against D614G strain and B.1.1.7 and B.1.617.2 variants	↓ 3.4 and 9.0 X in neutralizing B.1.1.7 and B.1.617.2
Mlcochova et al., 2021	BNT 162b2	10 vaccinee serum (after the 2^nd^ dose)	Pseudovirus neutralization against D614G strain and B.1.1.7 and B.1.617.2 variants	↓ 5.8 and 8.4 X in neutralizing B.1.1.7 and B.1.617.2
	Convalescent plasma	12 volunteers	Pseudovirus neutralization against D614G strain and B.1.1.7, B.1.351, and B.1.617.2 variants	↓ 2.3, 8.2, and 5.7 X in neutralizing B.1.1.7, B.1.351, and B.1.617.2
Muik et al., 2021	BNT 162b2	40 vaccinee serum (7 or 21 d after the 2^nd^ dose)	Neutralizing VSV pseudovirus (Wuhan strain and B.1.1.7 S mutants) entry in HEK-hACE2 cells	↓ (light reduction) in neutralizaing B.1.1.7
Planas et al., 2021	BNT 162b2	16 vaccinee serum (5 wks after the 2^nd^ dose)	S-Fuse neutralization assay against D614G strain and VOCs strains (B.1.1.7, B.1.351, and B.1.617.2)	↓ 0, 16, and 3 X in neutralizing B.1.1.7, B.1.351, and B.1.617.2
	AZD1222	20 vaccinee serum (4 wks after the 2^nd^ dose)		↓ 0, 9, and 5 X in neutralizing B.1.1.7, B.1.351, and B.1.617.2
	Convalescent plasma	26 convalesdent plasma (12 mo after the onset of symptons)		↓ 0, 4, and 4 X in neutralizing B.1.1.7, B.1.351, and B.1.617.2
Shen et al., 2021	mRNA-1273	28 vaccinee serum (28 d after the 2nd dose)		↓ 2X in neutralizing B.1.1.7
	NVX-CoV2373	28 vaccinee serum (2 wks after the 2nd dose)	Pseudovirus neutralization assay using D614G strain and B.1.1.7 variant	↓ 2 X in neutralizing B.1.1.7
	Convalescent plasma	15 convalesdent plasma (4- 9 mo after infection)		↓ 1.5 X in neutralizing B.1.1.7
Shinde et al., 2021	NVX-CoV2373	4,387 participants (2,199 vaccinated and 2,188 with placebo)	Clinical trial, phase 2a/b, in the South Africa	↓ Efficacy against B.1.351 (49.4%)
Supasa et al., 2021	BNT 162b2	25 vaccinee serum (7-17 d after the 2^nd^ dose)		↓ 3.3 X in neutralizing B.1.1.7
	AZD1222	10-15 vaccinee serum (14- 28 d after the 2^nd^ dose)	FRNT using Victoria and B.1.1.7 strains	↓ 2.1-2.5 X in neutralizing B.1.1.7
	Convalescent plasma	34 convalesdent plasma (4- 9 mo after infection)		↓ 2.9 X in neutralizing B.1.1.7
Wall et al., 2021	BNT 162b2	250 Individuals who worked at UCLH in UK and had received the vaccine (3 weeks, 6 and 12 weeks pos-vaccination)	RT-qPCR to exclude active infection; Blood was collected for serological assays including anti-spike IgG, IgM and live-virus neutralization; High-throughput live virus microneutralization assays	Reduction neutralizing antibodies activity against B.1.617.2 and B.1.351.
Wang et al., 2021	CoronaVac	25 vaccinee serum (2-3 wks after the 2^nd^ dose)		↓ 2 and 3.3 X in neutralizing B.1.1.7 and B.1.351
	BBIBP-CorV	25 vaccinee serum (2-3 wks after the 2^nd^ dose)	Pseudovirus neutralization against different strains (Wuhan-1 wild-type, D614G, B.1.1.7, and B.1.351)	↓ 0 and 2.5 X in neutralizing B.1.1.7 and B.1.351
	Convalescent plasma	34 convalescent plasma (5 mo. After infection)		↓ 1.1 and 2 X in neutralizing B.1.1.7 and B.1.351
Wibmer et al., 2021	Convalescent plasma	44 participants (mild-tomoderate and severe COVID-19)	Pseudovirus neutralization assay using D614G strain and B.1.351 variant	48% of the samples: loss of the neutralizing activity against B.1.351
Wu et al., 2021	mRNA-1273	28 vaccinee serum (one week after the 2^nd^ dose)	Pseudovirus neutralization assay using D614G strain and VOCs (B.1.1.7, B.1.351, and P.1 strains)	↓ 1.2, 6.4, 3.5 X in neutralizing B.1.1.7, B.1.351, and P.1
Zhou et al., 2021	BNT 162b2	25 vaccinee serum (4-17 d after the 2^nd^ dose)		↓ 7.6 X in neutralizing B.1.1.7
	AZD1222	25 vaccinee serum (28 d after the 2^nd^ dose)	FRNT using Victoria and B.1.351 strains	↓ 9 X in neutralizing B.1.1.7
	Convalescent plasma	34 convalesdent plasma (4- 9 mo after infection)		↓ 13.3 X in neutralizing B.1.351

FRNT, focus reduction neutralization test; PRNT, plaque reduction neutralization test; VSV, vesicular stomatitis virus.

## Variants of Concern and Potential Risk of New Pandemic Waves

From December 2019 until December 16^th^ 2021, there have been more than 271,376,000 COVID-19 cases and 5,325,969 COVID-19-related deaths (1.96% mortality rate) worldwide (World Health Organization, https://covid19.who.int). Moreover, approximately 3.4 million SARS-CoV-2 genome sequences have been submitted to the Global Initiative on Sharing All Influenza Data (GISAID; https://gisaid.org), which has detected more than 4,100 mutations in the *S* gene. About 1,200 of these mutations lead to amino acid substitutions, with 187 in the RBD of the S protein (Focosi and Maggi, 2021; Liu C. et al., 2021).

We performed a monthly analysis of the VOC emergence using the GISAID in several countries for one year (September 2020 to November 2021). The analysis of the epidemiological data of SARS-CoV-2 variants has several limitations, including a) a limited number of genome sequencing data from a particular country; b) samples from a particular group, city, or region that does not accurately represent the country; c) the virus’ behavior in a specific group, city or region; and d) data release delay (data were extracted and analyzed on September 2, 2021, but new sequencing genomes are continuously submitted and updated, especially in the last few months). However, it provides a general overview of specific variants globally and highlights some important points.

**Figure 4:**
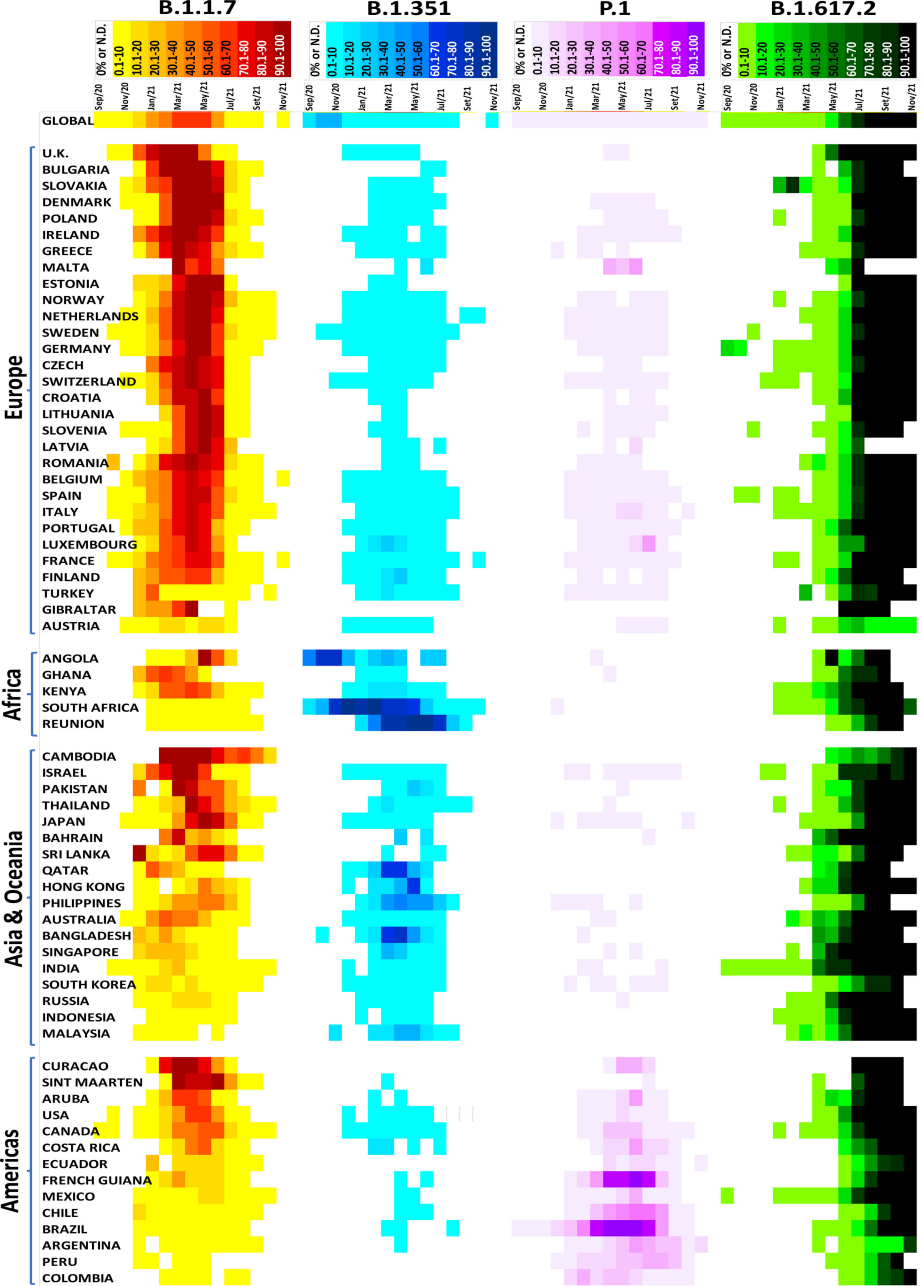
Distribution of the SARS-CoV-2 variants of concern in several countries. Data were analyzed from GISAID from September 2020 to November 2021 (https://www.gisaid.org/hcov19-variants/).

For example, after the emergence of the B.1.1.7 variant in the United Kingdom (September 2020), it rapidly spread to several countries across all continents (169 countries on Sep 2, 2021). In 34 of the 67 countries analyzed, the B.1.1.7 variant became highly predominant, present at rates greater than 80%; thus, demonstrating a clear selective advantage of this variant versus the original B.1 strain, which was the most prevalent strain at that moment in time (**Figure 4**). Additionally, in 12 of the 67 countries, this VOC was detected in 50.1 to 80% of the new monthly cases. In some countries, where other VOCs emerged before or even simultaneously, as in the case of B.1.351 in South Africa and Reunion and P.1 in Brazil, Chile, and French Guiana, the B.1.1.7 variant did not become predominant. This observation suggests that B.1.1.7 has no selective advantage over the B.1.351 and P.1 VOCs.

The B.1.351 variant emerged in South Africa in August 2020 and rapidly disseminated worldwide, reaching 111 countries as of September 2, 2021. Except for South Africa, Reunion, Angola, Philippines, Hong Kong, Bangladesh, and Qatar, the B.1.351 variant did not increase by more than 10% in most countries analyzed (**Figure 4**). A similar result was observed with the P.1 variant, which emerged in Brazil in December 2020. This variant disseminated at low rates (<10%) in 78 countries (Sep 2, 2021) but had a high prevalence in Brazil, Chile, and French Guiana (**Figure 4**).

The emergent B.1.617.2 variant appeared in India in October 2020. It has been dispersed throughout 147 countries (Sep 2, 2021) and has a high predominance rate in most analyzed countries. For example, more than 80% of COVID-19 cases were B.1.617.2-induced in 52 of the 67 countries analyzed; 4 had between 50.1 to 80%, and 11 had less than 50%. Notably, there is an increasing trend in the countries with fewer B.1.617.2-related cases. Currently, this variant is the most prevalent VOC, displaying rapid transmission and spread and indicative of selective advantages against other VOCs such as B.1.1.7, B.1.351, and P.1. It will likely become predominant worldwide. Fortunately, B.1.617.2’s high predominance has not increased the number of cases, hospitalizations or deaths, and the current vaccines effectively protect against all known VOCs.

## Perspectives and Concluding Remarks

Viral evolution is a constant process and can eventually improve “viral fitness” and selective adaptation. Emerging SARS-CoV-2 variants have posed challenges for authorities and scientists around the world. Although vaccines currently provide high protection against all VOCs, constant surveillance of vaccine efficacy is essential for combating the main SARS-CoV-2 strains and potentially new emerging variants.

The main concern is that a VOC can partially or completely evade the immune response, increasing reinfection of the individuals already infected by previous strains, limited protection induced by vaccination, and impaired efficacy of therapies based on monoclonal nAbs or convalescent plasma and consequently heightening the risk for future COVID-19 pandemic waves. Indeed, it has been proposed that the COVID-19 pandemic will persist for a long time with more mutations and emerging VOCs. Thus, actions must be undertaken to combat the COVID-19 pandemic and emerging VOCs. Below, we have highlighted seven key points that could prevent the rise of new SARS-CoV-2 variants:

1. *Rapid and massive worldwide vaccinations against COVID-19 to reduce new infections*. This point is based on the fact that slowing viral dissemination will reduce the probability of viral mutations and the emergence of new variants. However, vaccination campaigns are limited in some parts of the world. Thus, in these areas, strict public health measures and efficient strategies to stop or decrease virus transmission (e.g., face masks, frequent hand sanitation, social distancing, and other precautions) are the best defense against this virus.
2. *Constant and active global surveillance and identification of circulating and emerging VOCs and subsequent characterization*. Efficient monitoring systems will allow rapid detection, isolation, and response against new VOCs, avoiding uncontrolled dissemination and future pandemic waves.
3. *Determining vaccine and neutralizing antibody efficacy against VOCs*. If the vaccines do not present broad protection against the virus variants, periodic vaccine updates or redevelopment will be required, as occurs with the H1N1 vaccine. Other possibilities include developing new vaccines that induce nAbs against different variants by targeting highly conserved antigenic epitopes of the S protein and/or combining different vaccines or monoclonal Abs to target specific variants.
4. *Establish plasma repositories from individuals previously infected with different variants and immunized with different COVID-19 vaccines.* This point aims to rapidly determine the nAb activity against new VOCs and the potential for immune evasion. Determining the nAb titers and the period of protection induced by previous infection or vaccination is essential to determine further actions.
5. *Surveillance of reinfections, especially in already immunized or previously infected individuals*. This action could be a good strategy for assessing the potential immune evasion of new VOCs.
6. *Studies with combinations of available vaccines to improve efficacy and protection*. Monitoring the nAb levels for the S protein in fully vaccinated people can provide insights into protection since high levels of these antibodies seem to confer defense against emerging VOCs.
7. *Application of an additional booster vaccine dose to increase/prolong the neutralizing antibody titers over time*. This proposal is based on three points: a) high-risk groups, including immunocompromised and the elderly, present a reduced immune response following immunization; b) antibody titers decrease months after the complete vaccination schedule (14-21 days after the single dose Janssen vaccine or two doses of the other vaccines); and c) the emergence of VOCs may require high nAb titers for protection. Vaccine booster administration is already occurring in some countries, including the USA, Israel, and Brazil, especially in high-risk groups/individuals.

## Author Contributions

All authors attended the criteria to justify the authorship. Specifically, SH, DC, RC, and ED conceived the study. SH, TS, RG, LM, and TP-C elaborated the figures and tables, made the literature review and wrote the manuscript. DC, RC, and ED assisted the writing and revision of the manuscript. All authors have read, revised, and approved the final version of the submitted manuscript.

## Funding

The authors of this study are supported by grants from the São Paulo Research Foundation (FAPESP, Sao Paulo, SP, Brazil; 2018/09868-7 and 2021/00200-6), the Coordination for the Improvement of Higher Education Personnel (CAPES, Brasilia, Brazil), the National Council for Scientific and Technological Development (CNPq, Brasilia, Brazil), the John Simon Guggenheim Memorial Foundation (JSGMF, New York, NY, USA), and the Pro-Rectory of Post-Graduate and Research of the Cruzeiro do Sul University (PRPGP/Cruzeiro do Sul, São Paulo, SP, Brazil).

## Conflict of Interest

The authors declare that the research was conducted in the absence of any commercial or financial relationships that could be construed as a potential conflict of interest.

## Publisher’s Note

All claims expressed in this article are solely those of the authors and do not necessarily represent those of their affiliated organizations, or those of the publisher, the editors and the reviewers. Any product that may be evaluated in this article, or claim that may be made by its manufacturer, is not guaranteed or endorsed by the publisher.
